# A putative silencer variant in a spontaneous canine model of retinitis pigmentosa

**DOI:** 10.1371/journal.pgen.1008659

**Published:** 2020-03-09

**Authors:** Maria Kaukonen, Ileana B. Quintero, Abdul Kadir Mukarram, Marjo K. Hytönen, Saila Holopainen, Kaisa Wickström, Kaisa Kyöstilä, Meharji Arumilli, Sari Jalomäki, Carsten O. Daub, Juha Kere, Hannes Lohi, the DoGA Consortium

**Affiliations:** 1 Department of Veterinary Biosciences, University of Helsinki, Helsinki, Finland; 2 Department of Medical and Clinical Genetics, University of Helsinki, Helsinki, Finland; 3 Folkhälsan Research Center, Helsinki, Finland; 4 Department of Biosciences and Nutrition, Karolinska Institutet, Huddinge, Sweden; 5 Department of Equine and Small Animal Medicine, University of Helsinki, Helsinki, Finland; 6 Veterinary Clinic Kamu, Oulu, Finland; 7 Veterinary Clinic Malmin Eläinklinikka Apex, Helsinki, Finland; 8 Science for Life Laboratory, Karolinska Institutet, Stockholm, Sweden; 9 Stem Cells and Metabolism Research Program STEMM, University of Helsinki, Helsinki, Finland; HudsonAlpha Institute for Biotechnology, UNITED STATES

## Abstract

Retinitis pigmentosa (RP) is the leading cause of blindness with nearly two million people affected worldwide. Many genes have been implicated in RP, yet in 30–80% of the RP patients the genetic cause remains unknown. A similar phenotype, progressive retinal atrophy (PRA), affects many dog breeds including the Miniature Schnauzer. We performed clinical, genetic and functional experiments to identify the genetic cause of PRA in the breed. The age of onset and pattern of disease progression suggested that at least two forms of PRA, types 1 and 2 respectively, affect the breed, which was confirmed by genome-wide association study that implicated two distinct genomic loci in chromosomes 15 and X, respectively. Whole-genome sequencing revealed a fully segregating recessive regulatory variant in type 1 PRA. The associated variant has a very recent origin based on haplotype analysis and lies within a regulatory site with the predicted binding site of HAND1::TCF3 transcription factor complex. Luciferase assays suggested that mutated regulatory sequence increases expression. Case-control retinal expression comparison of six best HAND1::TCF3 target genes were analyzed with quantitative reverse-transcriptase PCR assay and indicated overexpression of *EDN2* and *COL9A2* in the affected retina. Defects in both *EDN2* and *COL9A2* have been previously associated with retinal degeneration. In summary, our study describes two genetically different forms of PRA and identifies a fully penetrant variant in type 1 form with a possible regulatory effect. This would be among the first reports of a regulatory variant in retinal degeneration in any species, and establishes a new spontaneous dog model to improve our understanding of retinal biology and gene regulation while the affected breed will benefit from a reliable genetic testing.

## Author summary

Retinitis pigmentosa (RP) is a blinding eye disease that affects nearly two million people worldwide. Several genes and variants have been associated with the disease, but still 30–80% of the patients lack genetic diagnosis. There is currently no standard treatment for RP, and much is expected from gene therapy. A similar disease, called progressive retinal atrophy (PRA), affects many dog breeds. We performed clinical, genetic and functional analyses to find the genetic cause for PRA in Miniature Schnauzers. We discovered two forms of PRA in the breed, named type 1 and 2, and show that they are genetically distinct as they map to different chromosomes, 15 and X, respectively. Further genetic, bioinformatic and functional analyses discovered a fully penetrant recessive variant in a putative silencer region for type 1 PRA. Silencer regions are important for gene regulation and we found that two of its predicted target genes, *EDN2* and *COL9A2*, were overexpressed in the retina of the affected dog. Defects in both *EDN2* and *COL9A2* have been associated with retinal degeneration. This study provides new insights to retinal biology while the genetic test guides better breeding choices.

## Introduction

Retinitis pigmentosa (RP) is a heterogeneous group of inherited retinopathies with varying genetic background and highly variable clinical consequences. RP is the leading cause of irreversible blindness in man with a worldwide prevalence of one in 4,000 people [1]. The disease first manifests as impaired vision in dim light (nyctalopia) resulting from progressive loss of the rod photoreceptor cells. As the disease progresses, complete blindness is expected due to cone photoreceptor degeneration accompanied by changes in the retinal pigment epithelium (RPE), the retinal vasculature, the glial cells and neurons of the inner retina. To date, 67 genes and loci have been implicated to nonsyndromic RP according to the Retinal Information Network RetNet (http://sph.uth.edu/retnet/) [2]. In the majority of cases, the mode of inheritance is autosomal recessive, although some autosomal dominant and X-linked RP exist [2]. Despite a large number of implicated genes and variants, 30–80% of the patients have RP of unknown genetic cause and thus many genes remain still to be discovered [3].

RP is incurable at the moment and much is expected from gene therapy to treat this disease. Different eye diseases have been favorite targets of gene therapy for several reasons, including relatively easy access to treat and monitor the target organ. A recent study described patient derived induced pluripotent stem cell (iPSC) treatment with clustered regularly interspersed short palindromic repeats (CRISPR/Cas9) to treat a RP affected patient with an X-linked point mutation in the *retinitis pigmentosa GTPase regulator (RPGR*) gene [4] in addition to other attempts to treat retinal dystrophies. As a known genetic cause of disease is obligatory to any gene therapy and still many RP related genes remain unknown, continuous attempts are needed to discover new causative variants and understand the underlying biology.

The domestic dog (*Canis familiaris*) has emerged as a powerful model to study human inherited diseases as its unique genetic architecture facilitates gene discoveries and many inherited diseases occur spontaneously in different breeds [5]. More than 220 genes have been implicated in various conditions over the past few years (www.OMIA.org) and the discovery rate is expected to remain high due to higher-resolution approaches and available genomic tools combined with the growing canine DNA biobanks worldwide [6,7]. Importantly, the dog presents an excellent model for human eye diseases, as the canine ocular globe bears more resemblance to the human eye, both anatomically and physiologically, than the eyes of the commonly used model organisms such as the mouse (*Mus musculus*) or the rabbit (*Oryctolagus cuniculus*) [8,9].

A similar phenotype to RP affects many dog breeds and is referred to as progressive retinal atrophy (PRA) [10,11]. Clinical findings in PRA include gradual loss of vision, tapetal hyperreflectivity and attenuation of the retinal blood vessels, which result from the degeneration of the photoreceptor layer in the retina–the pathology has great similarities to RP [10,12]. PRA affects over 100 dog breeds and currently 19 genes and 24 variants in 58 dog breeds have been implicated [13]. As with RP, the majority of the PRA related gene variants are autosomal recessive [13], but one dominant [14] and three X-linked variants [15–18] have been reported as well. In many dog breeds the causative variants have remained unknown, including the Miniature Schnauzers (MSs) in which the clinical findings have been previously described while the genetic background is undefined [19–22]. Recently, a genetic association was reported between a variant in *PPT1*, a well-known candidate gene for neuronal ceroid lipofuscinosis (NCL), and PRA in MS [23]. However, as the reported variant was incompletely penetrant (0.79) and as the affected MSs did not present with neurological signs seen in the NCL patients [23], the true causality of the variant remains elusive.

With a series of clinical, genetic, bioinformatic and biochemical experiments, we describe here two genetically different forms of retinal degeneration in the MS breed mapping to chromosomes 15 and X. We identify a novel fully penetrant recessive silencer variant in chromosome 15 with a possible gain-of-function effect resulting in overexpression of two retinal target genes, *EDN2* and *COL9A2*. Our results provide a new spontaneous dog model for retinal pathophysiology and insights to retinal biology and gene regulation. The affected breed will benefit from a genetic test for veterinary diagnostics and breeding advice.

## Results

### Ophthalmologic examinations reveal two types of retinopathy

To examine the PRA status of the study group of 85 MSs, careful eye examinations were performed with inclusion criteria for cases being bilateral PRA findings with tapetal hypo- and hyperreflectivity, vessel attenuation and pale optic nerve heads. The control dogs were free of any eye diseases at the age of seven years or older. This phenotyping approach resulted in a cohort of 18 cases and 67 controls. The clinical findings categorized the affected dogs to two distinctive groups: 12 dogs were diagnosed with severe PRA findings including total blindness before the age of five years (defined as type 1 in this study) while six of the cases had milder symptoms, slower progression with some visual capacity left at the time of examination and an average age of onset seven years. Of the type 1 affected dogs, five were females and seven males. Of the type 2 cases, only one was female while five were males, suggesting a possible sex-predisposition or X-linked mode of inheritance. Control group included 33 female and 34 male MSs.

**Fig 1:**
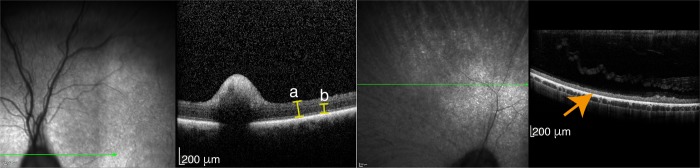
PRA confirmation by optical coherence tomography (OCT). OCT assessment of a healthy control (left hand side) and a four-year-old MS with severe type 1 PRA findings (right hand side). Whole retinal **(a)** and photoreceptor inner and outer segment and outer nuclear layer thickness **(b)** measurement showed total loss of the photoreceptor cell layers in the affected dog (orange arrow).

To evaluate the photoreceptor degeneration more precisely, OCT imaging was performed to a clinically blind, type 1 PRA case. The imaging showed complete loss of the photoreceptor layer (Fig 1) and thus confirmed the diagnosis. The clinical findings and the age of onset of the type 1 cases closely resemble the results of the previous study [22]. Type 2 dogs were not available for the OCT study.

### Genome-wide association study confirms two genetically distinctive types of PRA in MS

**Fig 2:**
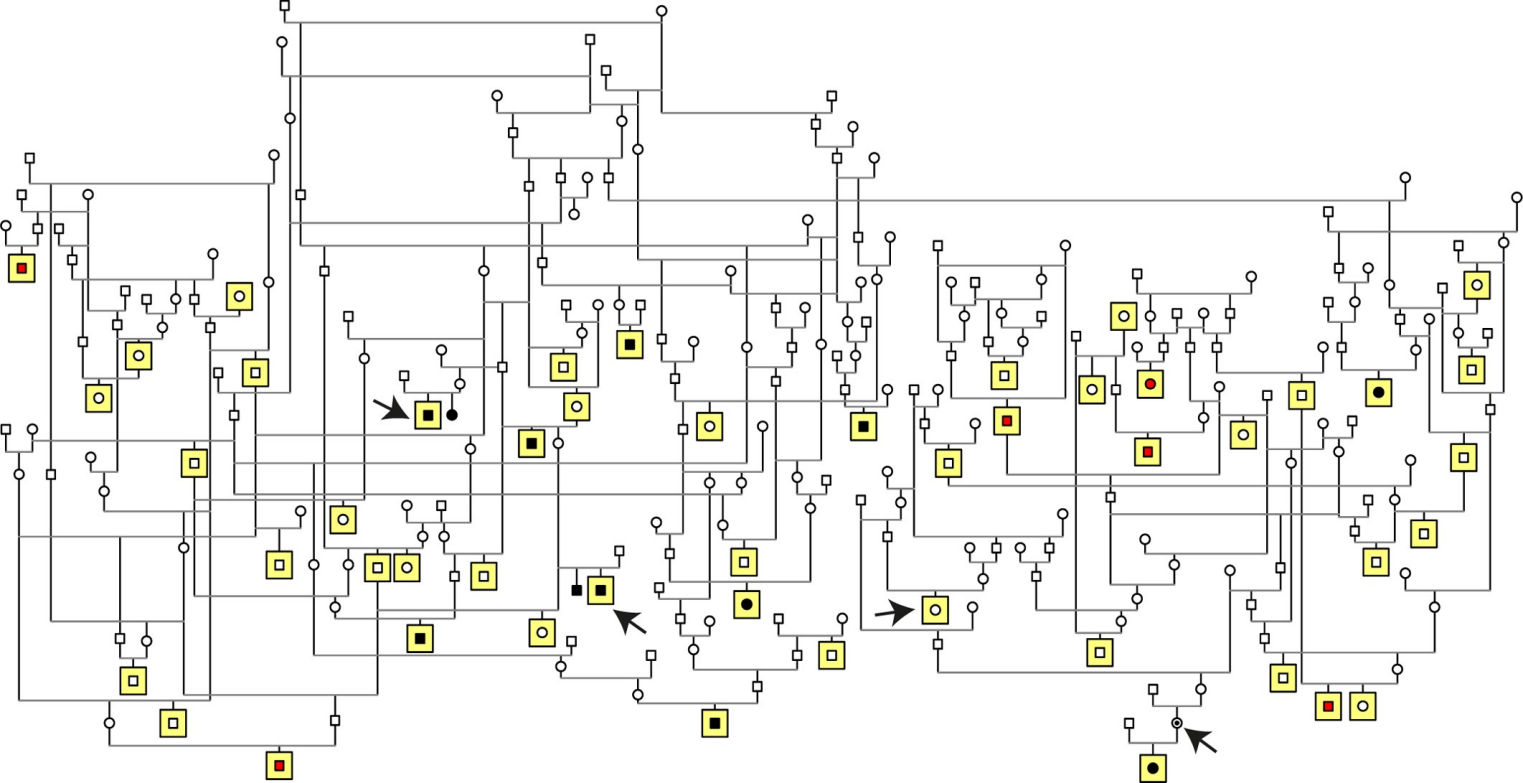
Pedigree of the study cohort. Pedigree analysis suggests a recessive mode of inheritance as affected dogs were born to unaffected parents. Squares indicate males and circles females. Individuals marked with black are type 1 cases, while type 2 cases are marked with red. Dogs with yellow background were genotyped and included in GWA study. Black arrows mark the whole-genome sequenced dogs.

A pedigree was drawn around the 18 affected dogs suggesting a recessive mode of inheritance, as affected dogs were born to unaffected parents (Fig 2). To map the disease associated chromosomal region, a genome-wide association study (GWAS) was conducted first with 16 cases (including 10 type 1 and six type 2 cases) and 33 controls. This analysis suggested a PRA locus on the canine chromosome 15 (p_raw_ = 4.70x10^-6^, p_genome_ = 0.19, λ = 1, S1A Fig). In-depth analysis of the genotypes in the locus showed that all of the type 1 cases shared a homozygous haplotype block of 7.2 Mb from 213,416 bp to 7,403,217 bp that was present neither in the 32 out of 33 control dogs nor in any of the type 2 cases. This result supports the hypothesis of the presence of two genetically distinct types of PRA in the MS breed. Interestingly, the homozygous risk haplotype block was present also in one control dog (S1B Fig). As all the other control dogs, this one had been eye examined at seven years of age without any clinical signs of PRA, suggesting either incomplete penetrance of the risk haplotype, or that the causative variant is recent and not all the risk haplotype carrying dogs have it.

**Fig 3:**
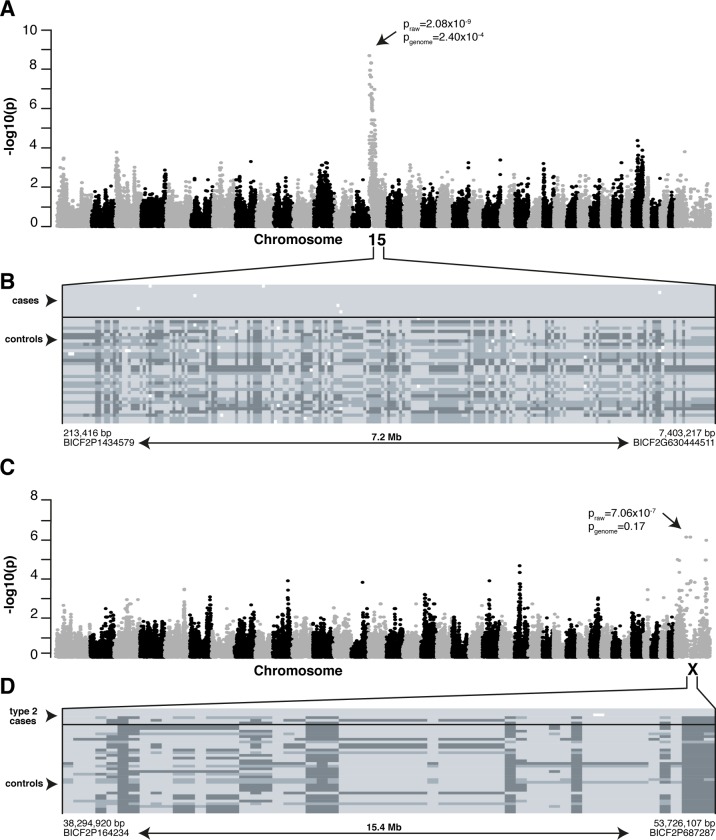
Mapping PRA loci in MSs. **(A)** Genome-wide comparison of allele frequencies in type 1 cases (n = 10) and controls (n = 33) indicated a locus on the CFA15 (p_raw_ = 2.08x10^-9^, p_genome_ = 2.40x10^-4^). **(B)** A shared homozygous haplotype block was seen in all the type 1 cases and one control and absent in 32/33 controls. Each row represents a single animal while genotypes at each SNP (columns) are marked with light (homozygous), dark (opposite homozygous) or intermediate (heterozygotes) grey. The critical region of 7.2 Mb spans from 213,416 bp to 7,403,217 bp and upper and lower limits of it were determined by appearance of heterozygous SNPs in the case dogs. **(C)** GWAS in type 2 cases (n = 6) and controls (n = 33) suggests a locus on CFAX (p_raw_ = 7.06x10^-7^, p_genome_ = 0.17). **(D)** A shared homo- or hemizygous haplotype block of 15.4 Mb (spanning nucleotides 38,294,920–53,726,107 bp) is present in 3 out of 6 type 2 cases and absent in the controls and the rest of the type 2 cases.

We then analyzed the genotyping data of the two PRA types separately. When comparing only the type 1 cases (n = 10) to the control dogs (n = 33), the same CFA15 locus and homozygous haplotype block was associated (Fig 3A and 3B), but with a stronger statistical significance (p_raw_ = 2.08x10^-9^, p_genome_ = 2.40x10^-4^, λ = 1.17). In addition, comparison of the type 2 cases (n = 6) and controls (n = 33) suggested an association to chromosome X (p_raw_ = 7.06x10^-7^, p_genome_ = 0.17, λ = 1.19), compatible with the suggested X-chromosomal sex pattern. In this locus, three of the six affected dogs shared a homo- or hemizygous haplotype block of 15.4 Mb from 38,294,920 bp to 53,726,107 bp that was absent in the controls and the rest of the type 2 cases (Fig 3C and 3D). As the number of type 2 cases was small, replication of the GWAS result in chromosome X in a larger sample cohort is needed to confirm the locus before further genetic analyses are performed. *RPGR*, the known canine PRA gene in CFAX, is not located in the preliminary locus [15–18].

### Whole-genome sequencing reveals four candidate variants

To identify the disease-causing variant in the associated chromosomal region for type 1 PRA in MSs, we performed whole-genome sequencing on three MSs including two type 1 cases with the risk haplotype and one control with opposite haplotype. In total, 467,778,694–512,026,483 reads were collected of which 99.5% were mapped to the reference sequence. Over 97% of the reference had >10X coverage while the mean read depths were 29–31. Filtering was done under recessive model, i.e., assuming that the cases were homozygous for the variant and at least 99% of the controls homozygous for the reference allele. The controls included one MS described above and 267 dogs of various breeds and without the studied phenotype (S1 Table). Of the controls, 141 were whole-genome sequenced and available also for a mobile element insertion (MEI) analysis, while 127 where exome-sequenced and therefore available only for analyzing single nucleotide variants and small insertions and deletions.

The two type 1 cases shared 2,149,016 common homozygous variants. After filtering against control dogs, 5,235 variants were left, of which 233 resided in the CFA15 locus (S2 Table). Of these, two were located in exons and two in splice sites (Table 1). These four variants were validated in the study cohort of 18 cases, including 12 type 1 PRA cases, and 67 control MSs. All the type 1 cases were homozygous for all the four variants, indicating association between the variants and the phenotype. However, there were also controls homozygous for the variants (n = 4 for the variant in *DLGAP3* and 2 for the other three variants), indicating either incomplete penetrance or that none of these variants are truly causative. We screened also the recently published variant in *PPT1* [23] in the cohort, although it was not caught by filtering. All type 1 cases were homozygous for the variant, while five type 2 cases were wild-type and one heterozygous. Of the 67 control dogs, 41 were wild-type, 22 heterozygous and four homozygous, accounting for an association between the type 1 disease and the *PPT1* variant (p = 1.6x10^-13^). However, this association was 10 to 100 times weaker than that with the other four variants in the same cohort (Table 1). MEI analysis revealed altogether 12 shared homozygous variants in the two cases in the locus, but none of them proved case-specific after filtering. No case-specific large structural variants were detected when analyzing the locus with IGV.

**Table 1:** Coding or splicing variants resulting from filtering the whole-genome sequencing data did not segregate with the phenotype.

Position (bp)^a^	Ref. allele	Alt. allele	Gene	Variant type	Variant (protein)	Transcript	p-value^b^
603,169	T	C	*C15H1orf50*	nonsynonymous SNV	p.M1V	XM_539558.4	2.1x10^-15
603,940	G	C	*P3H1*	splicing (c.477+13G = C)	-	XM_843477.4	2.1x10^-15
2,064,277	C	T	*SLFNL1*	splicing (c.852+7C = T)	-	XM_022427740.1	2.1x10^-15
7,077,805	CACCAC	del	*DLGAP3*	nonframeshift deletion	p.227_228del	XM_014119248.2	7.0x10^-14

^a^Coordinates refer to the CanFam 3.1 annotation.

^b^Association of the variant with type 1 PRA in initial study cohort of 85 MSs

### Sequencing an obligate carrier reveals a fully segregating regulatory variant

**Fig 4:**
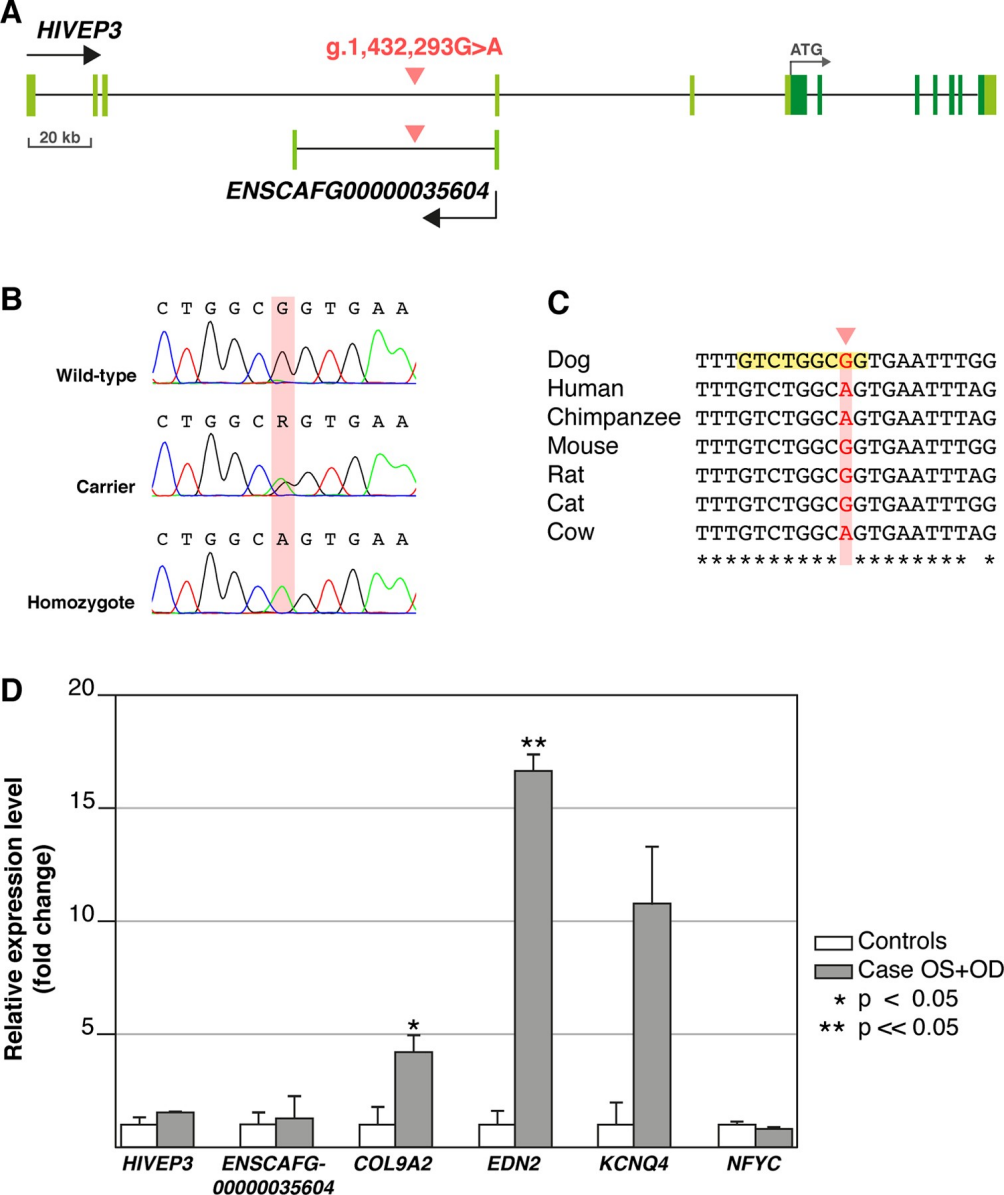
Schematic representation of the variant (g.1,432,293G>A) site and RT-qPCR results of HAND1::TCF3 target genes. **(A)** The *HIVEP3* and *ENSCAFG00000035604* gene structures with coding regions indicated in dark and non-coding in light green. The intronic variant (red) is located kilobases away of the exon-intron boundaries in both genes. **(B)** Sanger chromatograms showing the DNA sequence in wild-type, carrier and homozygous dogs with the mutated nucleotide marked with red background. **(C)** Alignment of different mammalian species showed that the sequence surrounding the variant site is highly conserved, but the variant nucleotide itself (highlighted in red) is not. JASPAR motif scanning indicated that TFBS of HAND1::TCF3 overlaps exactly the variant site (highlighted in yellow). **(D)** RT-qPCR of retinal samples from a type 1 affected MS and four control retinal samples wild-type for the variant and without the studied phenotype showed case-specific over-expression of *EDN2* and *COL9A2*. The comparative ΔΔC^T^ method was used to determine relative expression [24] and error bars are determined as standard deviation of the mean ΔC^T^.

The whole-genome sequencing results together with the fact that one of the control dogs in GWAS carried also the homozygous risk haplotype suggested that the causative variant for type 1 PRA in MSs is recent and not all the risk haplotype carrying dogs have it. To test this hypothesis, we performed whole-genome sequencing to an additional control MS, which was homozygous for all the four previously validated variants, but was unaffected still at the age of 10 years. In addition, this dog had a type 1 PRA affected offspring and thus was an obligate carrier for the causative variant (Fig 2). Altogether 456,191,937 reads were collected of which 99.3% were mapped to the reference sequence. Over 97% of the reference had >10X coverage and the mean read depth was 28. Filtering was performed as described above but by defining this sample as an obligate carrier. This approach reduced the case-specific variant list to five candidates including one intronic and four intergenic variants (Table 2). Three of the five variants appeared to be miscalled during validation by Sanger sequencing and IGV visualization. The two variants left, an intergenic 5-bp deletion (g.1,887,878_1,887,882del) and an intronic SNV (g.1,432,293G>A) in the transcription factor encoding the ortholog of *human immunodeficiency virus type I enhancer binding protein 3* gene (*HIVEP3*, Fig 4A and 4B) were validated in the study cohort of 18 cases and 67 controls. Again, all type 1 cases were homozygous for both of the variants. The intergenic 5-bp deletion was found to be homozygous in 12 of the 67 control dogs, thus excluding it from further analysis. The variant in *HIVEP3*, however, indicated complete penetrance as out of the 67 controls, 58 were wild-type, nine heterozygous and none homozygous. Thus, this variant segregated completely with the disease and the association with the type 1 phenotype was more than 10 billion times stronger than any other variants in the region (p = 4.0x10^-25^).

**Table 2:** Applying an obligate carrier to filtering decreased the number of variants to five, of which the intronic variant in *HIVEP3*/*ENSCAFG00000035604* had complete penetrance and strong association with the type 1 PRA in MSs.

Position (bp)^a^	Ref. allele	Alt. allele	Variant type	Gene
223,405	C	del	intergenic*	*ENSCAFG00000039839–ENSCAFG00000002484*
405,398	A	C	intergenic*	*ENSCAFG00000002491–ENSCAFG00000040650*
1,432,293	G	A	intronic	*HIVEP3*, *ENSCAFG00000035604*
1,887,878	AAAAT	del	intergenic	*ENSCAFG00000038329–SCMH1*
3,716,531	TTTCTT	del	intergenic*	*MACF1–NDUFS5*

^a^Coordinates refer to the CanFam 3.1 annotation.

*Miscalled, excluded by Sanger sequencing and IGV.

*HIVEP3* has been reported to be expressed ubiquitously, including the human retina, but it has not been implicated to retinal disease in any species [25]. The canine *HIVEP3* (XM_003431951.4) spans from 1,302,964 to 1,626,834 bp and includes 12 exons with protein coding sequence in exons 6–12 (Fig 4A). The associated variant in *HIVEP3* is located in the third intron: 105,943 bp downstream to the third and 32,572 bp upstream to the fourth untranslated exon. The surrounding sequence is ultraconserved (Fig 4B and 4C). There is also a predicted dog-specific long non-coding RNA (lncRNA) *ENSCAF00000035604* at the variant locus (Fig 4). This lncRNA is antisense to *HIVEP3* and consists of two exons and an intron. The variant is located 32,566 bp downstream of the first and 42,855 bp upstream to the second exon.

To further validate the intronic variant in *HIVEP3* in the MS breed, we genotyped all the available MS samples (n = 514) from our dog DNA bank, including three additional PRA cases and 118 dogs that were examined to have healthy eyes at the age of five or older. Of these, 456 (88.7%) were wild-type, 55 (10.7%) heterozygous and three (0.58%) homozygous for the variant. The identified three homozygotes were the PRA cases in the breed screening cohort and were clinically identical to type 1 cases as described above. These results in the breed screening cohort indicate again a complete penetrance of the intronic variant in *HIVEP3* and strong association with the type 1 phenotype (p = 8.7x10^-13^). Combining the results of the initial study cohort and the breed screening cohort resulted in a very strong association (p = 1.1x10^-42^) with the type 1 phenotype and was the only one of the validated variants with complete penetrance. The variant was not found from 735 genomes of different breeds available through The Dog Biomedical Variant Database Consortium dataset, which agrees with our hypothesis of a recent variant. No variants in the corresponding position in the human genome have been reported in the Genome Aggregation Database gnomAD [26].

### Associated variant is located within a putative silencer with HAND1::TCF3 transcription factor binding motif

The sequence surrounding the variant site is ultraconserved (Fig 4C), suggesting functional importance. The possible effect of the intronic variant on the splicing of the *HIVEP3* and lncRNA *ENSCAFG00000035604* transcripts was investigated by Sanger sequencing the retinal cDNAs from a case dog homozygous for the variant but indicated no changes compared to the wild-type transcript and thus ruled out splicing defects.

To further explore the functional significance of the associated variant, the syntenic site in the human genome was investigated, revealing that the variant is located in a peak of ENCODE DNaseI hypersensitivity cluster, which indicates an open chromatin environment and a possible regulatory site [27]. To gather further evidence, we next explored the JASPAR database to search for transcription factor binding sites (TFBSs) that could bind the sequence in the variant site. This search resulted in the discovery of a putative HAND1::TCF3 complex TFBS, overlapping exactly the variant location (Fig 4C).

**Fig 5:**
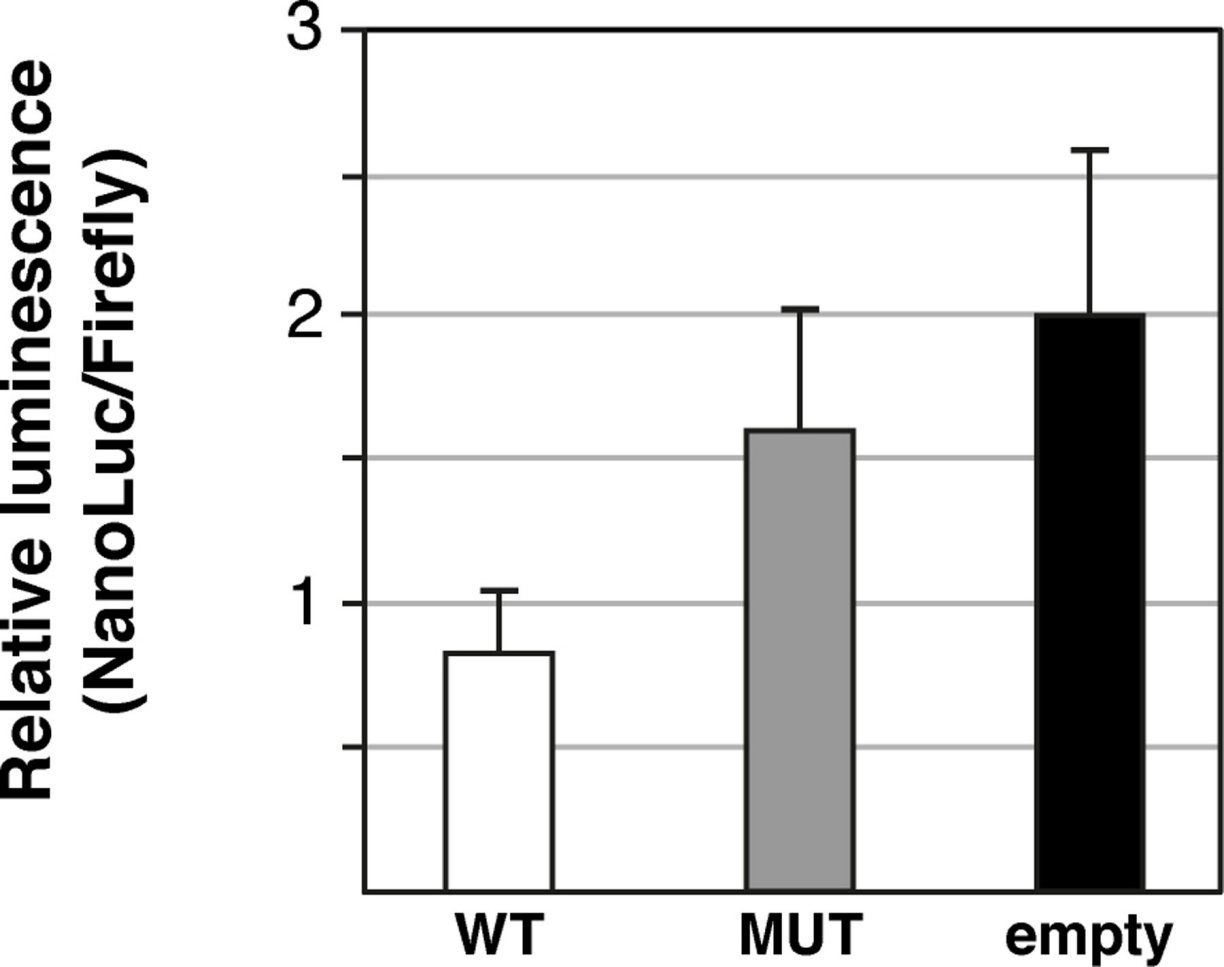
Reporter assay indicates the variant site might be a silencer. Dual luciferase assay with MDCK cells transfected with wild-type (WT), mutant (MT) and empty vectors indicated robust reduction in relative luciferase activity when comparing the wild-type constructs to empty vector, supporting the putative silencer activity (p < 0.05). Relative luciferase activity was significantly increased in mutant versus wild-type constructs (p < 0.05), indicating the type 1 PRA associated variant disrupts the putative silencer activity, but might not prevent it completely.

The possible regulatory function was studied by performing a dual luciferase reporter assay in MDCK cells transfected with wild-type, mutant, or empty vector constructs (S3 Table). The wild-type and mutant constructs contained 250 bp of upstream and downstream sequences from the variant site. Relative luciferase activity was significantly lower in cell lines transfected with the wild-type than with the empty vectors (p < 0.05, Fig 5), thus indicating silencer activity in the variant site. The PRA associated variant seems to disrupt the silencer activity as the cells transfected with the mutant constructs showed significantly higher reporter gene expression compared to the cells with the wild type constructs (p < 0.05, Fig 5).

### Retinal overexpression of *EDN2* and *COL9A2* in the affected dog

We next investigated whether the silencer variant has any effect on the expression of its target genes. Quantitative reverse-transcriptase PCR (RT-qPCR) with retinal samples from a type 1 PRA case and four unaffected dogs (wild-types for the variant) was performed for a selected set of target genes. As silencers can act from a distance, the presence of the HAND1::TCF3 motif was screened in the promoters of the flanking genes +/- 1.25 Mb from the variant site. Altogether 714 predicted HAND1::TCF3 binding sites were found in the promoter regions in 60 genes (S4 Table). Due to limited amount of available case-specific retinal RNA, six candidate genes were prioritized, including *HIVEP3*, *lncRNA (ENSCAFG00000035604)*, *COL9A2*, *EDN2*, *KCNQ4* and *NFYC*. Prioritization was based on the motif scanning score, known expression in retinal tissues in FANTOM5, Human Protein Atlas data [28,29] and retinal expression data from our STRT sequencing data from five dogs (S2 Fig and S4 Table). The RT-qPCR results indicated that retinal expression of *EDN2* was increased by over 16-fold (p = 0.0023) and *COL9A2* by over 4-fold (p = 0.049). Retinal expression of *KCNQ4* was increased by over 10-fold in the case retina, but the change was not statistically significant (p = 0.065), because of the large differences between retinal samples of the right and left eyes of the affected MS. Retinal *NFYC* expression was also not significantly changed (p>0.05). Given the lack of retinal tissues from additional affected and control MSs and to test the tissue-specificity of the regulatory sequence, we collected skin biopsies from three variant homozygotes, two carriers and three wild-type MSs and analyzed the potential differential expression of the 60 predicted target genes for HAND1::TCF3 by STRT. Out of the 60 genes, none were expressed in skin (S3 Fig), preventing us to compare the possible effect of the alternate genotypes on the transcript levels. It is likely that our candidate variant lies in a retina-specific regulatory region, which is supported also by the lack of extra-ocular phenotypes in the affected dogs.

## Discussion

RP is the leading cause of blindness in humans and nearly 70 genes have been implicated in various forms of the disease. However, the genetic origin remains still elusive in many cases [3]. We report here clinical and genetic characterization of a canine RP model (PRA), which provides new insights into the accumulating evidence of regulatory variants in the development of retinal disease.

The key findings of the study have several scientific and medical implications. We demonstrate the presence of at least two clinically and genetically different forms of PRA in the MS breed. The type 1 PRA with a homogeneous onset at 4 years of age mapped to chromosome 15, while the type 2 PRA, with an overrepresentation of affected males and a wider range of age of onset, mapped to chromosome X. While the tentative association in chromosome X needs to be replicated with additional cases, the association of the type 1 PRA in chromosome 15 was very strong. The origin of the causative variant was suspected to be very recent, since the homozygous risk haplotype was also present in unaffected dogs. Sequence analyses in the 7.2-Mb critical region associated with the type 1 PRA revealed a number of possibly pathogenic coding variants, however, all of them had incomplete penetrance (~70%). The incomplete penetrance of the many coding variants was consistent with the suspected recent origin of the actual causative variant. Indeed, the only fully penetrant variant located in the intron of *HIVEP3* transcription factor and a lncRNA *ENSCAFG00000035604*, however, kilobases away from any predicted splice sites. However, the expression of neither *HIVEP3* nor lncRNA (*ENSCAFG00000035604*) was significantly changed in the affected retina. Consistently, in silico analyses of the variant site predicted a binding site of the HAND1::TCF3 TF complex, suggesting a regulatory variant. Luciferase assays demonstrated an increased transcriptional activity of the reporter gene in the mutant recombinant, suggesting a gain-of-function variant in a silencer sequence. Silencer hypothesis was further supported by the observed overexpression of *EDN2* and *COL9A2* in the affected retina. Transcriptional regulation is often tissue-specific as observed also in this case with experiments in the skin biopsies and lack of extra-ocular phenotypes.

Altogether, these results suggest a loss-of-cell identity hypothesis: the identified regulatory variant affects a tissue-specific silencer and results in the upregulation of retinal gene expression, which in turn leads to the loss of cell- or tissue-specific identity and ultimately result in the degeneration of the photoreceptor cells. Similar mechanism has been reported for a regulatory variant in the GRHL2 gene in posterior polymorphous corneal dystrophy type 4 [30]. The proposed hypothesis is of particular interest, as the retinal phenotype in the MS breed was originally described a dysplastic disease leading to degeneration, and the clinical findings in our type 1 PRA affected MS group fully mimic the reported dysplasia [31]. However, further studies with larger canine retinal sample sets are needed to confirm the functional aspects of the variant. In addition, chromatin immunoprecipitation and EMSA gel shift assays are required to formally confirm the silencer function and its binding repressor(s), which would though need the development of dog-specific reagents.

Although our conclusions about the disease mechanisms are limited because of access to only one set of affected retinas from an 11-years-old type 1 affected MS, and should be therefore interpreted very cautiously, some discussion about the two target genes is warranted. *EDN2* encodes endothelin-2, which has been implicated in various cellular functions such as angiogenesis, cell migration, cell proliferation, and endothelial activation [32–36]. Overexpression of *Edn2* in the mouse retina inhibits retinal vascular development [37], which might also explain the etiology of the disease in MS type 1 cases as attenuation of retinal vessels is a hallmark feature in canine PRA [10] and was also observed in the cases here. However, *EDN2* overexpression might also function as a general stress signal from photoreceptor damage, and therefore it cannot be distinguished here, whether the observed overexpression of *EDN2* reflects the primary etiology of the retinal degeneration in type 1 PRA or the secondary stress reaction to the photoreceptor damage [32,38]. A loss-of-function variant in COL9A2 has been associated to autosomal recessive Stickler syndrome, in which vitreoretinal degeneration is one of the symptoms together with other ocular, auditory, skeletal and orofacial abnormalities [39].

Besides providing new insights into the possible disease mechanisms related to the underrepresented regulatory variants, our findings have important implication to the veterinary diagnostics and breeding programs. Recently, a complex structural variant in *PPT1* was proposed to cause PRA in MS [23]. This study, using lower resolution genotyping array, found a suggestive GWAS signal on the CFA15 with a ~5Mb critical interval that some, but not all, cases shared. Whole-genome sequencing approach identified the same intronic *HIVEP3* variant described here, but its potential functional role was not studied and the focus was turned to the complex structural variant in *PPT1* as it was homozygous in some of the affected dogs that were wild-type or heterozygous for the *HIVEP3* variant. Additional screening found also some of the controls to be homozygous for the *PPT1* variant, accounting for a penetrance of 79% [23]. In contrast, our data shows that the breed is affected with multiple genetically distinct forms of PRA and that the causative variant has such a recent origin that not all risk haplotype-carrying dogs have it. This leads to the identification of many candidate variants in the critical interval that seem to, misleadingly, associate with the phenotype, but have incomplete penetrance. In contrast, in our cohort, the predicted silencer variant located in the *HIVEP3* intron, has complete penetrance and 10 billion times stronger association with the type 1 PRA as compared with the PPT1 variant in our study cohort. Our study provides a proper marker for genetic testing avoiding unnecessary confusion within the breeding community.

To summarize, we have identified two forms of retinal degeneration in a dog breed and showed that these phenotypes are distinct both clinically and genetically. We mapped two new loci and identified a type 1 disease-specific recessive variant in a putative silencer located in predicted HAND1::TCF3 TFBS with two of its target genes overexpressed in the affected retina sample. These results establish the MS dog breed as a spontaneous large animal model to study human RP and provide a new potential regulatory variant to understand the retina in health and disease.

## Materials and methods

### Ethics statement

All dogs used in this study were privately owned pet dogs. EDTA blood samples (3 ml) from all the dogs, skin biopsies from eight dogs and retinal samples from six dogs (euthanized because of severe diseases, described in detail below) donated to research were collected under the permission of animal ethical committee of County Administrative Board of Southern Finland (ESAVI/343/04.10.07/2016) and all experiments were performed in accordance with relevant guidelines and regulations and with owners’ written consent.

### Study cohort

This study included altogether 599 privately owned purebred MSs, including a discovery cohort of 85 dogs and a population screening cohort of 514 dogs. The discovery cohort included 18 cases and 67 control dogs, which all were eye examined by veterinary ophthalmologists board-certified by the European College of veterinary ophthalmologists. The eye examination included basic neuro-ophthalmic examination, slit-lamp biomicroscopy to evaluate the adnexa and anterior segment and indirect ophthalmoscopy to examine the posterior parts of the eye. Topical tropicamide (Oftan Tropicamid 1%, Santen, Tampere, Finland) was used to achieve mydriasis. Optical coherence tomography, OCT, (Heidelberg’s Spectralis, Heidelberg Engineering GmbH, Heidelberg, Germany) was performed to one PRA affected MS. Inclusion criteria for cases were bilateral severe PRA findings. Controls did not show any symptoms of eye disease and were minimum of seven years old at the time of the examination. Population based variant screening was performed in 514 MSs of which three were affected with type 1 PRA and 118 were eye examined healthy at the age five or older by veterinary ophthalmologists as described above. EDTA blood samples (3 ml) from all the dogs and tissue samples from six euthanized dogs donated to research were collected under the permission of animal ethical committee of County Administrative Board of Southern Finland (ESAVI/343/04.10.07/2016) and all experiments were performed in accordance with relevant guidelines and regulations. Genomic DNA was extracted from the white blood cells using a semi-automated Chemagen extraction robot (PerkinElmer Chemagen Technologie GmbH, Baeswieler, Germany) according to the manufacturer’s instructions. DNA concentrations were measured using Qubit fluorometer (Thermo Fisher Scientific, Waltham, Massachusetts, USA) and Nanodrop ND-1000 UV/Vis Spectrophotometer (Nanodrop technologies, Wilmington, Delaware, USA) and samples were stored at –20°C.

### Genome-wide association study

A genome-wide association study, GWAS, was performed using Illumina’s CanineHD BeadChip arrays with 172,963 markers (San Diego, CA, USA). Genotyping was performed in GeneSeek Laboratory (Neogen Genomics, Lincoln, NE, USA) to 16 cases and 33 controls. After quality control procedures, only SNPs which conformed to Hardy-Weinberg expectations P<0.0001, had >95% genotyping rate and minor allele frequency of >5% were included in the analysis, resulting in the total number of 102,661 SNPs when analyzing all the cases and controls and 100,501 and 102,286 when comparing separately the type 1 cases and type 2 cases to controls, respectively. The differences in allele frequency between cases and controls were calculated using the PLINK 1.07 software [40]. Corrected empirical p-values were calculated with 100,000 permutations and genomic control adjusted significance values based on the estimation of the inflation factor.

### Whole-genome sequencing

Whole genome sequencing was performed to two cases and two controls using the Illumina HiSeq X ultra-high-throughput sequencing platform with 30X target coverage (paired-end reads, 2x 150 bp) (Novogene Bioinformatics Institute, Beijing, China). Mapping the reads to the dog reference genome (assembly CanFam 3.1) was performed using the Burrows-Wheeler Aligner (BWA) version 0.7.15 [41]. The reads were sorted and duplicate reads marked using the Picard tools (http://sourceforge.net/projects/picard). Post-alignment processing includes local realignment around known INDELs and base quality scores recalibration to reduce erroneous variant calls [42]. Variant calling was done using the HaplotypeCaller in Genome Analysis Tool Kit GATK version 3.7. Functional annotation of the variants was done in ANNOVAR using Ensembl, NCBI and Broad gene databases. Mobile element insertions were detected in the three whole-genome sequenced samples using the Mobile Element Locator Tool (MELT) [43]. The reference sequences of the transposons for MEI discovery were retrieved from Repbase database [44]. The detected variants were stored in Genotype Query Tools (GQT) [45] variant database and variant filtering was performed using our in-house pipeline assuming recessive mode of inheritance, i.e. the cases were homozygous for the alternative alleles. The variant data from whole-exome (n = 127) and whole-genome sequenced (n = 140) dogs without the studied phenotype and from various breeds were used in filtering as controls (S1 Table). A maximum of 1% of the controls were allowed to be heterozygotes of the alternative allele, while the rest were set to be homozygous for the reference allele.

### Sanger sequencing and TaqMan genotyping

Sanger sequencing was utilized to perform segregation analyses for the variants found in filtering the whole-genome sequencing data. Segregation analysis of the *PPT1* structural variant was performed utilizing the consecutive markers (g.2,872,023_2,872,024del and g.2,872,103G>A) confirmed to be consistent with the actual complex structural variant in the original study [23] and which was also validated in our whole-genome sequenced MS samples. All other variants were directly sequenced. Primers (S5 Table) were designed using the Primer3 program [46] and PCR products amplified using Biotools DNA Polymerase (Biotools B&M Labs, S.A., Valle de Tobalina, Madrid, Spain). The PCR products were treated with exonuclease I and shrimp alkaline phosphatase and capillary sequenced in the Institute for Molecular Medicine Finland (Helsinki, Finland). The Sanger sequence data were analyzed using Sequencher 5.1 (Gene Codes Corporation, Ann Arbor, MI, USA) and Unipro UGENE 1.31.1 (UniPro, Novosibirsk, Russia). Variant screening in the MS breed cohort with 514 dogs was performed using Custom TaqMan SNP Genotyping Assay (S5 Table) chemistry (ThermoFisher Scientific, Waltham, MA, USA) and CFX96 Touch Real-Time PCR Detection System (BioRad, Hercules, CA, USA).

### *In-silico* analysis of transcription factor binding sites and target gene prioritization

The UCSC liftOver tool was utilized to convert the corresponding variant site in the human genome version GRCh38. The overlapping TFBS motif (HAND1::TCF3) was obtained from the JASPAR database [47]. Motif scanning was done using “searchSeq” function of TFBSTools package (v.1.20) under R programming language [48] on the +/-500 bp regions surrounding the TSSs of CanFam3.1 Ensembl genes version 94 [49]. Target gene prioritization was performed based on motif scanning score and confirming retinal expression in canine STRT sequencing data and in the FANTOM5 [28] and the Human Protein Atlas [29] databases. STRT RNA-sequencing was performed to retinal samples collected from five dogs: a 3-year-old female Swedish Elkhound, 5- and 6-year-old male Rottweilers, a 6-year-old female Finnish Lapphund and a 6-year-old male Border Collie that were all wild-type for the type 1 PRA associated variant and without the studied phenotype. The dogs were privately owned pets. The Swedish Elkhound and the Border Collie were euthanized because of epilepsy, the Rottweilers due to gastrointestinal disease and the Finnish Lapphund due to intervertebral hernia. Samples were collected within 15 minutes after death and treated with Invitrogen RNAlater Stabilization Solution (Thermo Fisher Scientific, Waltham, Massachusetts, USA) for 24 hours and stored in –80°C. RNA was extracted using the RNeasy Mini Kit (Qiagen, Venlo, Netherlands) according to manufacturer’s instructions. Integrity of RNA was evaluated with Agilent 2100 Bioanalyzer (Agilent Technologies, Santa Clara, California, USA) and concentration measured with Qubit fluorometer (Thermo Fisher Scientific, Waltham, Massachusetts, USA) and Nanodrop ND-1000 UV/Vis Spectrophotometer (Nanodrop technologies, Wilmington, Delaware, USA). 20 ng of RNA was used for multiplex 48 sample RNA-seq libraries, which were prepared using modified STRT method with unique molecular identifiers [50,51]. The modifications included longer UMI’s of 8 bp, addition of spike-in ERCC control RNA for normalization of expression, and use of Globin lock method [52] with LNA-primers for canine alpha- and betaglobin genes. The libraries were sequenced with Illumina NextSeq 500, High Output (75 cycles). Reads were mapped to CanFam3.1 genome annotation using HISAT1 mapper version 2.1.0 [53]. Gene level quantification was done using featureCounts version 1.6.2 [54] and the counts were then normalized to the library size and transcript length. The expression levels of 60 flanking genes (S4 Table) was then visualized using the ComplexHeatmap package [55].

### Dual luciferase reporter assay

To verify the predicted silencer role and to study whether the found variant has an effect on its function, constructs containing the wild-type and mutated canine sequence (CanFam 3.1, 250 bp +/- of the variant site) were generated (S3 Table) and cloned into the pNL3.2[*NlucP*/minP] NanoLuc luciferase vector (Promega, Madison, WI, US). The pGL4.54[luc2/TK] firefly luciferase was used as a co-transfection control vector (Promega, Madison, WI, US). For transfection assay, 3 x 10^5^ MDCK cells were seeded per well in 96 well cell culture microplate with white walls (CellStar, Greiner bio-one, Germany) 24h prior to transfection, using Gibco Advanced MEM medium (Thermo Fisher Scientific, Waltham, Massachusetts, USA) supplemented with 10% FBS, 100 U penicillin/streptomycin and Glutamax. Cell transfection was performed using Lipofectamine3000 (Invitrogen, Carlsbad, US) according to manufacturer instructions. The co-transfection was performed using 10 ng wild-type, mutated or empty experimental vector, 10 ng control vector and 90 ng carrier DNA per well. Luciferase activities were measured after 48 h using the Nano-Glo® Dual-Luciferase® Reporter Assay System (Promega, Madison, WI, US) according to the manufacturer’s instructions and luminescence was detected using the Enspire 2300 instrument (PerkinElmer Chemagen Technologie GmbH, Baeswieler, Germany). Luminescence values were normalized by calculating the ratio between experimental (NanoLuc) and control (firefly) reporter. Statistical significance of the results was assessed by one-way ANOVA and Bonferroni approach as post-hoc test and p-value < 0.05 was considered significant. The assays were performed twice with 4 to 6 replicates per treatment.

### Target gene expression analysis in retina and skin biopsies

Retinal samples from both eyes were collected from a 11-year old male MS affected with type 1 PRA and homozygous for the intronic variant in *HIVEP3*. Control retinal samples were collected from a 2-year-old female German Pinscher, a 3-year-old female East-European Shepherd, a 5-year old male Smooth Collie and a 5-year-old male Rottweiler that were all wild-type for the variant and without the studied phenotype. The dogs were privately owned pets. The MS was euthanized because of congestive heart failure, the Rottweiler due to inflammatory bowel disease and the German Pinscher, Smooth Collie and the East-European Shepherd due to behavioral problems. Samples were collected and treated as were the ones used for the STRT experiment described above. Reverse transcription from RNA to cDNA was performed to equal amounts of sample RNA with High Capacity RNA-to-cDNA Kit (Applied Biosystems, Waltham, Massachusetts, USA). Primer3 program [46] was used to design the primers (S4 Table) for the six target genes (*HIVEP3*, *ENSCAFG00000035604*, *COL9A2*, *EDN2*, *KCNQ4*, *NFYC*) and the two house-keeping genes (*GAPDH* and *YWHAZ*), that were used as normalization controls. Forward and reverse primers were positioned in different exons to avoid genomic DNA contamination. Primer efficiencies were determined from a seven-point dilution series and the comparative ΔΔC^T^ method was used to determine relative expression [24]. Real-time quantitative PCR (RT-qPCR) was performed with SsoAdvanced Universal SYBR Green Supermix (BioRad Hercules, CA, USA) and the CFX96 Touch Real-Time PCR Detection System (BioRad, Hercules, CA, USA) according to manufacturer’s instructions. Three technical replicates were used for all reactions. Error bars were determined as standard deviation of the mean ΔC^T^.

Skin biopsies were collected from eight MSs including three PRA affected variant homozygotes, two carriers and three wild-type dogs. Samples were taken from the dorsal neck region with punch biopsy technique using local infiltration anesthesia with 2% lidocaine (Orion Pharma, Espoo, Finland) and stored in RNAlater Stabilization Solution (Invitrogen, Thermo Fischer Scientific, Waltham, Massachusetts, USA). RNA was extracted using the NucleoSpin RNA kit (Macherey-Nagel Gmbh, Germany) according to manufacturer’s instructions. RNA concentration and integrity were measured as described above.

## Supporting information

S1 Fig Initial mapping approach for PRA locus in MSs. **(A)** Genome-wide comparison of allele frequencies in all cases regardless of the disease type (n = 16) and controls (n = 33) suggested a locus on the CFA15 (p_raw_ = 4.70x10^-6^, p_genome_ = 0.19). **(B)** A shared homozygous haplotype block was seen in all the type 1 cases and absent in all the type 2 cases and in 32/33 controls, indicating the two clinically distinct types are indeed genetically different. Interestingly, the risk haplotype was also present in one of the confirmed controls, indicating either incomplete penetrance of the risk haplotype, or that the causative variant is recent and not all the risk haplotype carrying dogs have it. Each row represents a single animal while genotypes at each SNP (columns) are marked with light (homozygous), dark (opposite homozygous) or intermediate (heterozygotes) grey. The critical region of 7.2 Mb spans from 213,416 bp to 7,403,217 bp and upper and lower limits of it were determined by appearance of heterozygous SNPs in the case dogs.
(TIF)

S2 Fig Heat map from the canine retinal STRT sequencing experiment. Retinal samples from five dogs wild-type for the type 1 PRA associated variant and without the studied phenotype were STRT sequenced to examine retinal gene expression of the 60 target genes for HAND1::TCF3 TFBS. Columns indicate individual samples (SE = Swedish Elkhound, RW = Rottweiler, FL = Finnish Lapphund, BC = Border Collie), rows each target gene. Darker green color indicates stronger expression in the retina (TPM = transcripts per million). The six genes prioritized by several factors for RT-qPCR were all expressed in the canine retina (ENSCAFG00000002576, HIVEP3; ENSCAFG00000002827, NFYC; ENSCAFG00000035604, lincRNA; ENSCAFG00000002579, EDN2; ENSCAFG00000002997, COL9A2; ENSCAFG00000002814, KCNQ4).
(PDF)

S3 Fig Heat map from the canine skin STRT sequencing experiment. Skin biopsies from eight MS including two heterozygotes (orange), three variant homozygotes (red) and three wild-type dogs (green) were collected and STRT sequenced to examine potential differences in the expression levels of the 60 target genes. As a result, none of the target genes were expressed in the skin.
(TIF)

S1 Table Filtering the whole-genome sequencing data: Controls. Control dogs used for filtering the whole-genome sequencing data (numbers per breed).
(XLSX)

S2 Table Filtering the whole-genome sequencing data: Results. Filtering the whole-genome data from two type 1 PRA cases against 268 controls resulted in 233 variants.
(XLSX)

S3 Table Dual Luciferase reporter assay. The construct sequences used for MDCK cell transfections.
(PDF)

S4 Table Motif search results. Motif search found 714 predicted HAND1::TCF3 binding sites (Sheet1) in the promoter regions (+/- 500 bp TSS) of 60 genes (Sheet2) flanking the variant site. The STRT-based retinal expression levels of the 60 genes in the ten dogs used in the DoGA project (Sheet 3).
(XLSX)

S5 Table Primer sequences. Primer sequences for Sanger sequencing, RT-qPCR and Taqman assay.
(XLSX)
