# Supplementary figures and images for: A putative silencer variant in a spontaneous canine model of retinitis pigmentosa
Source: PLoS Genet. 2020 Mar 9;16(3):e1008659. doi: 10.1371/journal.pgen.1008659 (PMC7082071; doi:10.1371/journal.pgen.1008659)

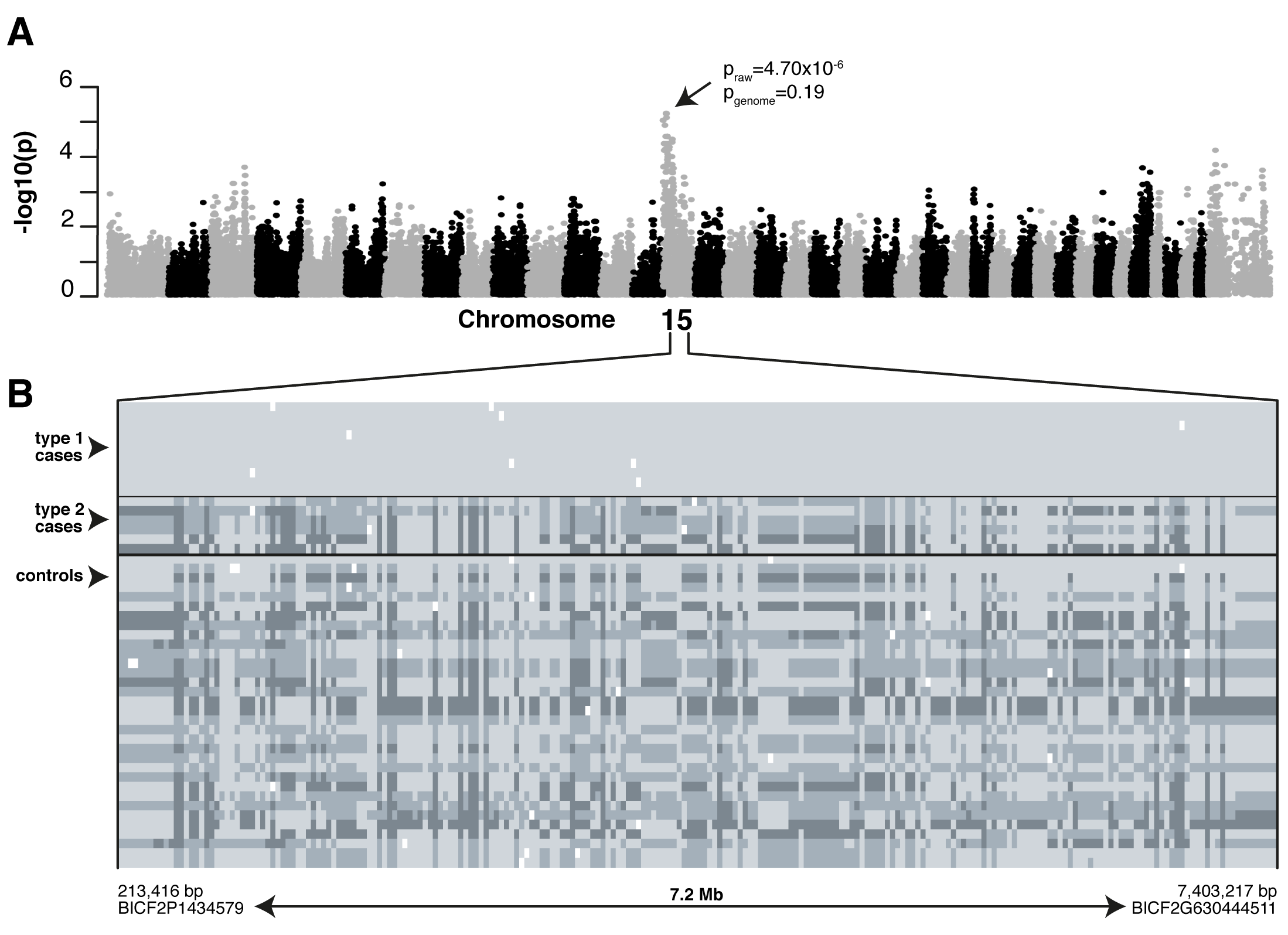

Supplement: S1 Fig — (A) Genome-wide comparison of allele frequencies in all cases regardless of the disease type (n = 16) and controls (n = 33) suggested a locus on the CFA15 (praw = 4.70x10^-6^, pgenome = 0.19). (B) A shared homozygous haplotype block was seen in all the type 1 cases and absent in all the type 2 cases and in 32/33 controls, indicating the two clinically distinct types are indeed genetically different. Interestingly, the risk haplotype was also present in one of the confirmed controls, indicating either incomplete penetrance of the risk haplotype, or that the causative variant is recent and not all the risk haplotype carrying dogs have it. Each row represents a single animal while genotypes at each SNP (columns) are marked with light (homozygous), dark (opposite homozygous) or intermediate (heterozygotes) grey. The critical region of 7.2 Mb spans from 213,416 bp to 7,403,217 bp and upper and lower limits of it were determined by appearance of heterozygous SNPs in the case dogs. (TIF) [file pgen.1008659.s001.tif]

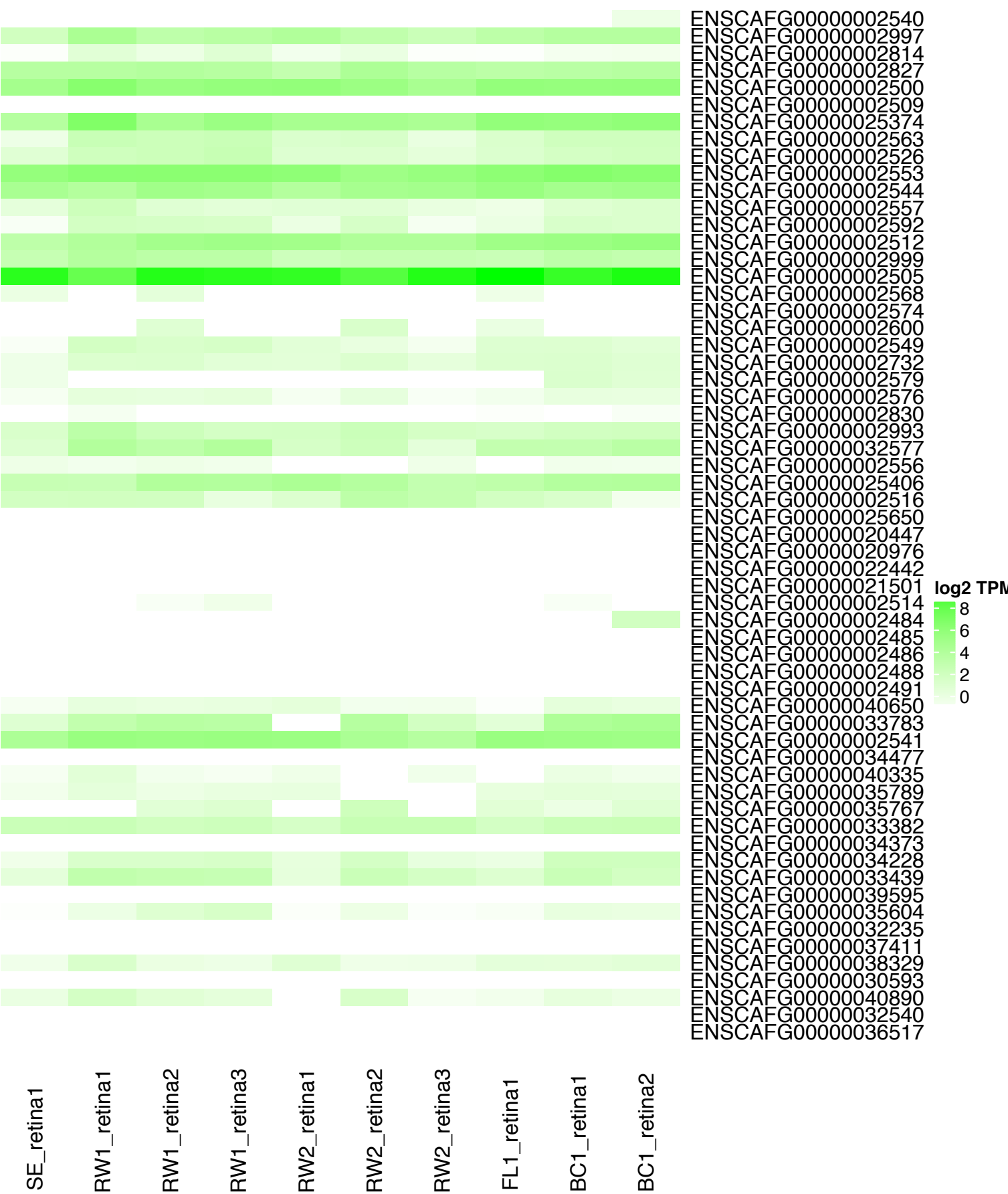

Supplement: S2 Fig — Retinal samples from five dogs wild-type for the type 1 PRA associated variant and without the studied phenotype were STRT sequenced to examine retinal gene expression of the 60 target genes for HAND1::TCF3 TFBS. Columns indicate individual samples (SE = Swedish Elkhound, RW = Rottweiler, FL = Finnish Lapphund, BC = Border Collie), rows each target gene. Darker green color indicates stronger expression in the retina (TPM = transcripts per million). The six genes prioritized by several factors for RT-qPCR were all expressed in the canine retina (ENSCAFG00000002576, HIVEP3; ENSCAFG00000002827, NFYC; ENSCAFG00000035604, lincRNA; ENSCAFG00000002579, EDN2; ENSCAFG00000002997, COL9A2; ENSCAFG00000002814, KCNQ4). (PDF) [file pgen.1008659.s002.pdf]

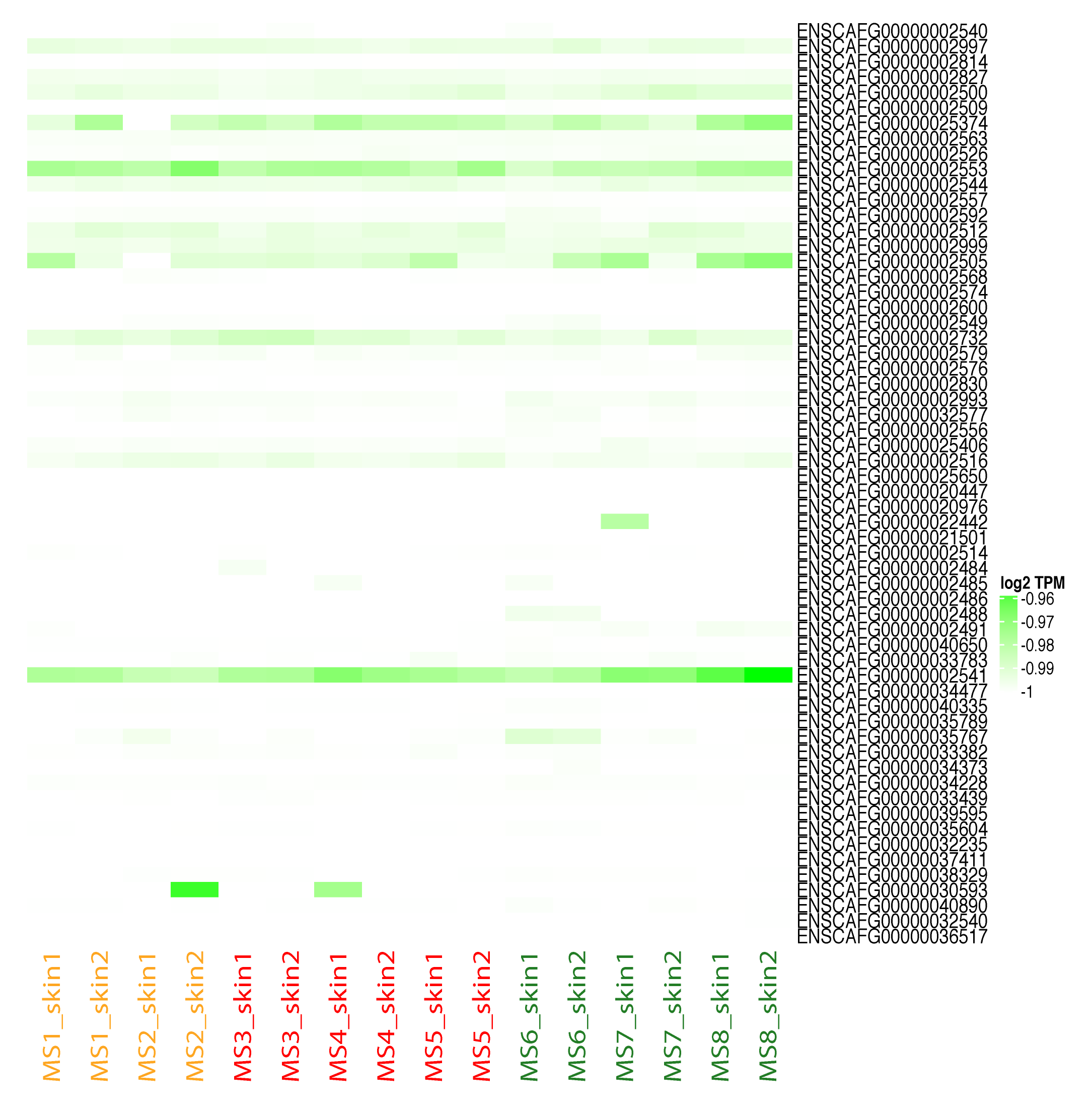

Supplement: S3 Fig — Skin biopsies from eight MS including two heterozygotes (orange), three variant homozygotes (red) and three wild-type dogs (green) were collected and STRT sequenced to examine potential differences in the expression levels of the 60 target genes. As a result, none of the target genes were expressed in the skin. (TIF) [file pgen.1008659.s003.tif]
