# Supplementary material for: A putative silencer variant in a spontaneous canine model of retinitis pigmentosa
Source: PLoS Genet. 2020 Mar 9;16(3):e1008659. doi: 10.1371/journal.pgen.1008659 (PMC7082071; doi:10.1371/journal.pgen.1008659)
Supplement: S3 Table — The construct sequences used for MDCK cell transfections. (PDF) [file pgen.1008659.s006.pdf]

**Name**	**Sequence**
Wild-type	GGTACCATTGAACGGGAAAGGTGGCTCTTCAGAGGCAGCTGTCTCCCCCGC<br>ACAGCCGACAAATCATTGTTTTAATGAGTTGTCACTTGGACAATGGGGCTA<br>AGTCTTGGCCCTGATTCAGTGCTCTTGCCTTCTCCCCACCCGCACCCCCTCCT<br>TCTATGCACCCCCCCTCCCCGGCTTGCTTGCTTCTCTCTCTAGCCTCCACTTA<br>CTGGAAATGAAATCTCTCAGCGCTTGAATGGCAGGGAGGGAGGGAGGCG<br>CCCAGACAAAGAGAAGGCTTTGTCTGGC**G**GTGAATTTGGATGGCAGGTGG<br>GTGGCGGGGAGCACACAGACTTCCTCCATCCGCCTGTCACGGAGCTCGCCT<br>AGTGTCGGAGGAGGGGAGCTGGGTTCAGTCCTCTGGCAACGCCTGGCTTC<br>CAAATGATGGGTTTGCAGAGAGAGCCCCAGGCTGCCAGCCCCTGGTAGGA<br>GGAGGCCAGAGCCGTGGCAGGTCGCTCGGTGGGGGCAGGGGTGTGAGTG<br>GGGCTGCAAGCTCCCACTCGAGGGCAGCCAGGGAGCCAGATGAGGCTGG<br>AAGCTT
Mutant	GGTACCATTGAACGGGAAAGGTGGCTCTTCAGAGGCAGCTGTCTCCCCCGC<br>ACAGCCGACAAATCATTGTTTTAATGAGTTGTCACTTGGACAATGGGGCTA<br>AGTCTTGGCCCTGATTCAGTGCTCTTGCCTTCTCCCCACCCGCACCCCCTCCT<br>TCTATGCACCCCCCCTCCCCGGCTTGCTTGCTTCTCTCTCTAGCCTCCACTTA<br>CTGGAAATGAAATCTCTCAGCGCTTGAATGGCAGGGAGGGAGGGAGGCG<br>CCCAGACAAAGAGAAGGCTTTGTCTGGC**A**GTGAATTTGGATGGCAGGTGG<br>GTGGCGGGGAGCACACAGACTTCCTCCATCCGCCTGTCACGGAGCTCGCCT<br>AGTGTCGGAGGAGGGGAGCTGGGTTCAGTCCTCTGGCAACGCCTGGCTTC<br>CAAATGATGGGTTTGCAGAGAGAGCCCCAGGCTGCCAGCCCCTGGTAGGA<br>GGAGGCCAGAGCCGTGGCAGGTCGCTCGGTGGGGGCAGGGGTGTGAGTG<br>GGGCTGCAAGCTCCCACTCGAGGGCAGCCAGGGAGCCAGATGAGGCTGG<br>AAGCTT
